# Diagnostic Approach to Seronegative Autoimmune-Mediated Rhabdomyolysis: A Case Report

**DOI:** 10.7759/cureus.113239

**Published:** 2026-07-23

**Authors:** Cyrus Behzadi, Nathaniel Neavling, Rahul Kurapati

**Affiliations:** 1 Medicine, SUNY Downstate Health Sciences University, New York, USA; 2 Neurology, Lenox Hill Hospital, New York, USA; 3 Internal Medicine, Lenox Hill Hospital, New York, USA

**Keywords:** autoimmune necrotizing myopathy, muscle mri, rhabdomyolysis, seronegative, statin

## Abstract

Rhabdomyolysis is a potentially life-threatening condition caused by acute skeletal muscle injury leading to myocyte destruction and release of intracellular contents. While trauma and medications are common triggers, autoimmune-mediated necrotizing myopathies are rare. Diagnostic evaluation becomes challenging in seronegative cases. We report a 51-year-old man with multiple cardiovascular comorbidities presenting with one week of progressive proximal muscle weakness, severe back pain, and dark urine. Initial labs revealed a creatine kinase (CK) of 24,700 U/L, transaminitis, and acute kidney injury. Urinalysis showed heme positivity without red blood cells, consistent with myoglobinuria. MRI of the thighs demonstrated muscle edema. Common precipitants, including trauma, exertion, infection, toxins, thyroid disease, and statin-associated antibody-mediated myopathy, were excluded by history, serology, and laboratory workup. Despite negative autoimmune panels (anti-nuclear antibody (ANA), anti-hydroxy-3-methylglutaryl-CoA reductase (HMGCR) antibody, and anti-Jo1), the clinical course and imaging supported a diagnosis of seronegative immune-mediated necrotizing myopathy. This case highlights an unusual presentation of rhabdomyolysis with associated symptoms of proximal muscle weakness and a slow improvement in CK levels despite fluids in a patient seronegative for autoimmune markers.

## Introduction

Rhabdomyolysis results from skeletal muscle injury leading to release of creatine kinase (CK), myoglobin, and intracellular electrolytes into the circulation [1]. While trauma, exertion, medications, and toxins are the most common causes, autoimmune-mediated necrotizing myopathies (IMNM) represent a rare but important etiology, particularly in patients with prior statin exposure [2]. Antibody assays, such as anti-HMGCR and anti-SRP, can facilitate diagnosis; however, a subset of patients remain seronegative. We present a case of severe rhabdomyolysis with acute kidney injury in which diagnostic reasoning suggested seronegative autoimmune-mediated myopathy.

## Case presentation

A 51-year-old man with a history of type 2 diabetes mellitus (HbA1c 9.6%), hyperlipidemia on long-term rosuvastatin and ezetimibe, hypertension, coronary artery disease status post coronary artery bypass grafting (CABG) and multiple stents, and heart failure with recovered ejection fraction of 50% presented with one week of progressive diffuse back pain, bilateral proximal weakness, and dark urine. He denied trauma, exertion, immobilization, seizures, drug use, alcohol, or recent viral illness. Medication regimen at presentation: metformin 500 mg BID, rosuvastatin 40 mg, Zetia 10 mg, Entresto 24-26 mg, aspirin 81 mg, ticagrelor 90 mg, and metoprolol 50 mg. 

On arrival, he was hemodynamically stable and afebrile. Neurological exam revealed 4/5 hip flexion strength bilaterally with preserved distal strength. The remainder of the exam was otherwise unremarkable. 

Initial laboratory testing

Significant values include CK 24,700 U/L, creatinine 2.37 mg/dL (baseline ~1), BUN 31 mg/dL, AST 584 U/L, ALT 638 U/L, and glucose 347 mg/dL (Table 1). Urinalysis showed heme positivity with absent RBCs and granular casts. 

**Table 1:** Patient's initial laboratory values

Test name	Result	Normal range
Creatine kinase (CK)	24,700 U/L	30-200 U/L (males)
		30-135 U/L (females)
Creatinine	2.37 mg/dL	0.3-1.2 mg/dL
		(baseline ~1 mg/dL)
Blood urea nitrogen (BUN)	31 mg/dL	9.5-46.3 mg/dL
Aspartate aminotransferase (AST)	584 U/L	12-60 U/L
Alanine aminotransferase (ALT)	638 U/L	11.2-48.0 U/L (males)
		4.3-37 U/L (females)
Glucose	347 mg/dL	65-125 mg/dL
Urinalysis - heme	Positive	Negative
Urinalysis - red blood cells	Absent	0-2 per high-power field
Urinalysis - casts	Granular casts present	None or occasional hyaline casts
Thyroid-stimulating hormone (TSH)	Normal	0.4-4.0 mIU/L (approximate)
Antinuclear antibody (ANA)	Negative	Negative
Anti-HMG-CoA reductase antibody	Negative	Negative
Anti-histidyl-tRNA synthetase (anti-Jo-1)	Negative	Negative
Toxicology screen	Negative	Negative

Imaging

**Figure 1:**
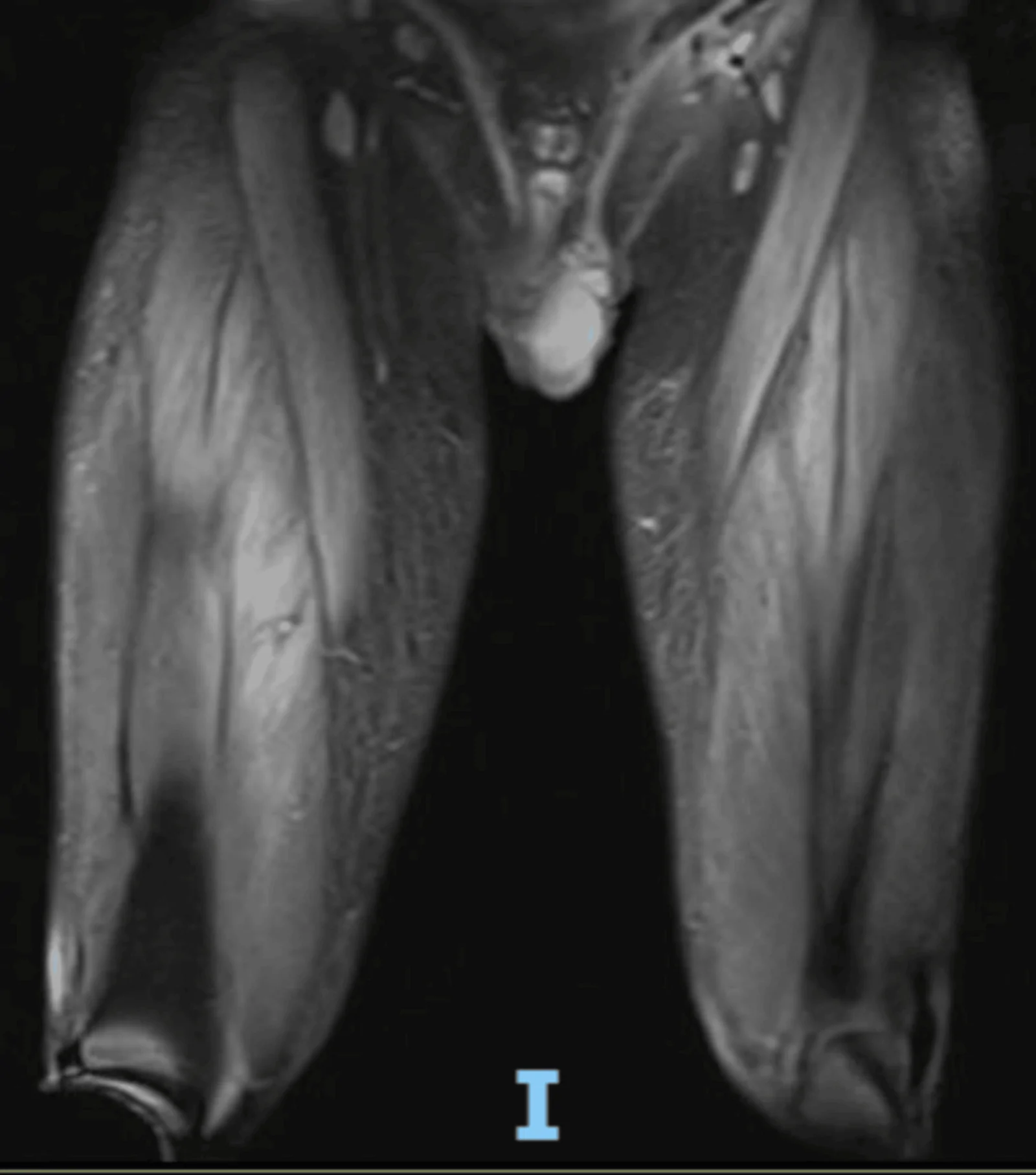
MRI lower-extremity non-joint with/without IV contrast MRI lower-extremity non-joint with/without IV contrast showing diffuse high short TI inversion recovery (STIR) signal with associated enhancement involving bilateral thigh musculature, most prominent in the mid to distal portion of the right sartorius muscle, right mid semimembranosus muscle, and left rectus femoris.

MRI of the thighs demonstrated diffuse STIR hyperintensity with contrast enhancement involving multiple proximal muscle groups, consistent with myositis (Figure 1). Lumbar spine MRI showed degenerative disc changes without evidence of radiculopathy. 

The patient was managed with aggressive IV fluids, strict input/output monitoring, and discontinuation of statins. Renal function stabilized, and CK initially trended downward on hospital day two. However, the patient’s CK continued to uptrend on day three of his hospitalization, progressing from an initial 24,700 to 26,200 despite fluids (Table 2). The continued elevation of CK suggested an autoimmune inflammatory etiology of the rhabdomyolysis and prompted further investigation. Eventually, after one week of hospitalization, he improved clinically and was discharged with outpatient neurology and rheumatology follow-up for EMG and muscle biopsy. 

**Table 2:** Patient's creatine kinase (CK) levels throughout his admission

Date	HD1 08:00	HD2 16:00	HD2 22:00	HD3 11:00	HD4 08:00	HD4 12:00	HD4 16:00	HD5 12:00	HD6 08:00	HD7 09:00	HD8 06:00
CK (U/L)	24700	18100	26200	18693	16983	17906	13713	9634	5885	3618	1875

The patient’s CK and creatinine eventually improved with supportive management. He was discharged in stable condition with outpatient follow-up for EMG and muscle biopsy to confirm suspected seronegative immune-mediated necrotizing myopathy. 

On the outpatient follow-up, the patient’s CK normalized and inflammatory markers were negative. Definitive diagnosis of immune-mediated necrotizing myopathy is dependent on muscle biopsy, which is still pending for the patient to undergo. However, the dramatic improvement with statin cessation and supportive care, initial clinical presentation, and MRI findings remain highly suspicious for immune-mediated necrotizing myopathy. 

## Discussion

This case illustrates the diagnostic challenges of autoimmune-mediated rhabdomyolysis in the absence of confirmatory serologies. Statin-associated IMNM is often antibody-positive; however, approximately two-thirds of cases may lack detectable anti-HMGCR antibodies [3]. Diagnosis must therefore integrate clinical presentation, imaging, and exclusion of alternative etiologies. Along with cessation of any offending agents and supportive care, treatment for IMNM typically consists of immunosuppressive therapy, often high-dose glucocorticoids in combination with steroid-sparing immunosuppressive agents. Statin-induced IMNM treatment can include adjunctive intravenous immunoglobulins (IVIG) as well.

Concomitant use of statin and ticagrelor has been shown to induce rhabdomyolysis and therefore remains a likely differential for our patient's presentation [4]. However, the patient was maintained on DAPT therapy throughout his hospitalization. While this does not necessarily exclude the statin-tigacrelor combination as a rhabdo etiology, the continuation of one of the proposed offending agents does not correlate with the patient's rapid clinical improvement. 

While statins are a well-recognized cause of both toxic and immune-mediated myopathies, long-term therapy does not preclude the development of late autoimmune complications [5,6]. Furthermore, clinical features of toxic statin myopathy involve a rapid resolution of elevated CK once statins are held. However, in our patient, his persistent initial elevation and eventual slow resolution of CK levels increase the likelihood of immune-mediated myopathy. This is further supported by MRI findings showing asymmetric edema of the semimembranosus and rectus femoris muscles, which is more consistent with IMNM compared to the symmetrical, pan-compartmental edema associated with statin toxicity [7]. 

## Conclusions

Seronegative immune-mediated necrotizing myopathy should be considered in patients presenting with severe rhabdomyolysis and proximal weakness when common causes are excluded, and MRI suggests inflammatory myopathy. Accurate diagnosis of IMNM is essential, as management varies from traditional rhabdomyolysis. Further research is needed to determine alternative biomarkers in patients with seronegative IMNM, with some data suggesting anti-signal recognition particle antibody (anti-SRP) as a potential supplement. 

This case report highlights the diagnostic approach to an atypical presentation of rhabdomyolysis. It is important to emphasize that our findings for this study did not include a definitive muscle biopsy; given the clinical features of this case, we maintain a high clinical suspicion of inflammatory myopathy.
